# Angelman syndrome in Poland: current diagnosis and therapy status—the caregiver perspective: a questionnaire study

**DOI:** 10.1186/s13023-024-03292-w

**Published:** 2024-08-22

**Authors:** Agata Suleja, Katarzyna Milska-Musa, Łukasz Przysło, Marzena Bednarczyk, Marcin Kostecki, Dominik Cysewski, Paweł Matryba, Anna Rozensztrauch, Michał Dwornik, Marcin Opacki, Robert Śmigiel, Kacper Łukasiewicz

**Affiliations:** 1 Faculty of Medicine, Medical University of Silesia, Katowice, Poland; 2 Angelman Syndrome Project, PROT sp. z o.o., Bialystok, Poland; 3 Division of Quality of Life Research, Department of Psychology, Faculty of Health Sciences with the Institute of Maritime and Tropical Medicine, Medical University of Gdansk, Gdansk, Poland; 4 Department of Developmental Neurology and Epileptology, Research Institute of Polish Mother’s Memorial Hospital, Lodz, Poland; 5 Department of Propaedeutics of Obstetrics, Faculty of Health Sciences in Katowice, Medical University of Silesia, Katowice, Poland; 6 Clinical Research Centre, Medical University of Bialystok, Bialystok, Poland; 7 Department of Immunology, Faculty of Medicine, Medical University of Warsaw, Warsaw, Poland; 8 Division of Family and Pediatric Nursing, Department of Nursing and Obstetrics, Faculty of Health Sciences, Wroclaw Medical University, Wroclaw, Poland; 9 Department of Pediatrics, Endocrinology, Diabetology and Metabolic Diseases, Faculty of Medicine, Wroclaw Medical University, Wroclaw, Poland; 10 Centre of Medical Rehabilitation and Osteopathy REHApunkt, Warsaw, Poland; 11 Experimental Linguistics Lab, Faculty of Modern Languages, University of Warsaw, Warsaw, Poland; 12 Experimental Medicine Centre, Medical University of Bialystok, Bialystok, Poland; 13 Department of Psychiatry, Faculty of Medicine with the Division of Dentistry and Division of Medical Education In English, Medical University of Bialystok, Bialystok, Poland; 14 Uniwersyteckie Centrum Chorób Rzadkich, Wroclaw Medical University, Wroclaw, Poland

**Keywords:** Angelman syndrome, Rase diseases, Healthcare organisation, Genetic testing

## Abstract

**Background:**

Angelman syndrome (AS) is a rare neurodevelopmental disease caused by imprinting disorders that impede the production of the ubiquitin E3A ligase protein (UBE3A). AS affects multiple systems, with the main symptoms including epilepsy, psychomotor disorders and speech development disorders. To date, no study has been conducted in the Polish population to verify the condition's diagnosis and treatment process.

**Results:**

Seventy patients with the median age of 60 months were included into the analysis. 80% of patients were diagnosed with deletion, 19.9% with a mutation of UBE3A gene, 4.3% with paternal uniparental disomy (UPD) and 2.8% with an imprinting defect. The mean age of first symptoms was 5 months, while the mean age of diagnosis was 29 months (earliest in deletion group at 23 months), and the median duration of diagnosis process was 7 months. The average time to a clinical geneticist appointment was 3 months. 37.9% of the patients initially received a different diagnosis. Epileptic seizures were present in 88.6% of the individuals. 98.6% of the studied group were under care of a pediatric neurologist, 47.1% of a gastroenterologist. A ketogenic diet was used in 7.1% of patients. Caregivers identified finding a specialist suitable for AS patients and access to genetic testing as the biggest problems.

**Conclusions:**

The care of patients with AS in Poland is carried out according to the European and world standards, however there is an impeded access to clinical geneticist, and the knowledge about rare diseases among primary healthcare physicians could be improved. Moreover, access to AS care specialists and coordination of care is limited. There is a need for creation a specialized centers and databases for AS patients.

## Background

Angelman syndrome (AS) is, along with Prader–Willi syndrome (PWS), a typical example of a disease caused by imprinting disorders, which are part of epigenetic disorders. It is a severe neurodevelopmental condition causing impairment of multiple systems. Thus, coordinated patient care and collaboration between different specialists are so important in the care of patients with AS [1]. AS is also an example of a rare genetic disease, where cooperation between health professionals such as doctors and therapists, parents, and patient organizations is crucial [2].

The main symptoms presented by patients with AS are feeding disorders, sucking problems in infancy, delayed psychomotor development, absent or limited speech development, gait disorders, tremors in the limbs, ataxia, and most significantly, intellectual disability, epilepsy, as well as microcephaly appearing with age. Symptoms are caused by a disruption in the production of the ubiquitin E3A ligase protein (UBE3A) by reduced or absent expression of the *UBE3A* gene [1, 3–5].

AS is a rare disease, making the development of precise management protocols challenging. However, international, worldwide registries exist to compile patient data, facilitating guidelines creation and improving patient care quality [6, 7]. Despite Poland having a population of approximately 38 million, there is no such registry. It impedes the establishment of organized approach and standardized care for patients with AS. Additionally, knowledge of rare diseases in general is lacking among Polish physicians, necessitating additional training [8].

The main aim of the study was to determine which recommendations have actually been implemented into everyday practice and are used in the diagnosis and treatment of the Polish population of patients with AS. Moreover, we have compared our results with data from other countries and identified which aspects of Polish healthcare system need improvement. A survey was conducted among caregivers of individuals with AS to verify what the diagnosis and treatment process look like from their perspective.

### Epidemiology

AS is diagnosed in 1/12000 to 1/20000 individuals in the general population [1]. Unfortunately, there is no precise data regarding the prevalence of the syndrome in the Polish population. Due to the lack of an authoritative register of people with rare diseases, including AS in Poland, the actual estimation of the size of this patient population is a significant challenge. Some countries, including Denmark, established relevant registries for example Danish National Patient Registry (NPR) and the Danish Cytogenetic Central Registry (DCCR) [9].Otherwise, data about patients is gathered by devoted centers, such as the Dutch ENCORE Expertise Centre for AS in Rotterdam [10].

## Methods

In order to initially characterize the Polish AS population, an anonymous survey was conducted among parents and caregivers of people with AS concerning both the process and problems that occur during diagnosis and treatment of AS in Poland. The survey consisted of 46 questions—single-choice and multiple-choice, as well as open-text questions [Table 1]. Bioethical clearance to conduct the study was obtained via the approval of the Bioethics Committee at the Medical University of Wrocław (no. 125/2023). The survey clearance was shared online in September 2022. The inclusion criteria for the study were: (1) physician-confirmed diagnosis of AS; and (2) the course of the entire diagnostic and therapeutic process in Poland. The questionnaire was completed by 75 individuals; five of them did not meet the inclusion criteria; responses from 70 people were included in the final analyses.

**Table 1:** Questions—the diagnosis and treatment of Angelman Syndrome in Poland

#	Question	Answer type	Choice options
1	What is the age of the affected individual? (in months)	[Short answer]	–
2	What is the height of the affected individual? (in cm)	[Short answer]	–
3	What is the weight of the affected individual? (in kg)	[Short answer]	–
4	What were the first concerning symptoms that motivated you, as caregivers, to seek further diagnosis?	[Multiple choice]	a. Problems with suckling b. Delays in the development of motor skills c. Delays in intellectual development d. Speech impairment or no speech at all e. Problems with balance f. Characteristic behavior, such as laughter that is inappropriate in the given situation g. Microcephalia h. Epileptic seizures i. Characteristic EEG readouts j. Abnormal muscle tension k. Other
5	At what age did the first concerning symptoms appear? (age in months)	[Short answer]	–
6	At what age did the diagnosis of Angelman syndrome begin? That is, when did the first visit to a dedicated specialist physician or the first stay at a hospital ward occur? (age in months)	[Short answer]	–
7	What motivated the diagnosis? Who observed the concerning symptoms?	[Single choice]	a. Caregivers at home b. A physician during a medical checkup c. The child had been hospitalized and attending physicians began to suspect AS d. Other
8	What did the diagnosis entail (what tests were performed up to this point)?	[Multiple choice]	a. Medical history, a physical examination b. The mother’s and the child’s HRT karyotype c. The FISH or aCGH test (MicroMatrices) d. Methylation test e. Molecular analysis of the UBE3A gene f. Other
9	How long was the diagnosis? (i.e. from which to which month of the patient’s life?)	[Short answer]	–
10	Did the diagnosis involve paid medical services (i.e. ones not covered by state medical insurance)? If so, please state the estimated total cost of these services (down to the nearest 100 PLN)	[Short answer]	–
11	Was any other diagnosis stated prior to that of AS? (If so, please indicate which)	[Short answer]	–
12	How long was the wait before the first scheduled visit to a designated clinical geneticist? (in months)	[Short answer]	–
13	At what age was AS finally diagnosed? (age in months)	[Short answer]	–
14	Which genetic background factor was determined during diagnosis?	[Single choice]	a. Deletion b. Mutation of the UBE3A gene c. Uniparental paternal disomy (UPD) d. Imprinting defect e. Clinical diagnosis (based on symptoms) f. Other
15	Is the affected individual under the supervision of a child neurologist/neurologist? (checkups on at least a semi-annual basis)	[Single choice]	a. Yes b. No
16	Did/does the affected individual experience epileptic seizures?	[Single choice]	a. Yes b. No c. Other
17	If so, then at what age did the first epileptic seizure occur? (age in months)	[Short answer]	–
18	What medications were administered as treatment for the epileptic seizures? Please mark all that have been used (brand names given in brackets)	[Multiple choice]	a. Clobazam (Frisium) b. Levetiracetam (Cezarius, Keppra, Levebon, Normeg, Polkepram, Trund, Vetira) c. Clonazepam (Clonazepamum) d. Valproic acid (Convulex, Depakine, ValproLEK) e. Phenobarbital (Luminalum, Bellergot) f. Ethosuximide (Petinimid) g. Felbamate (Felbatol, Taloxa) h. Phenytoin (Epanutin parenteral, Phenytoinum WZF) i. Gabapentin (Epigapent, Gabagamma 100, Gabapentin Aurovitas, Gabapentin TEVA, Neurontin, Symleptic) j. Carbamazepine (Amizepin, Finlepsin, Neurotop, Tegretol) k. Lamotrigine (Epitrigine, Lamilept, Lamitrin, Lamotrix, Symla) l. Lorazepam (Lorabex, Lorafen, Lorazepam Orion, Temelor) m. Oxcarbazepine (Karbagen, Oxcarbazepin NeuroPharma, Oxepilax, Trileptal) n. Pregabalin (Pregabalin Zentiva) o. Topiramate (Epitoram, Etopro, Oritop, Topamax, Topamax, Topiramat Bluefish, Toramat) p. Vigabatrin (Sabril) q. Zonisamide (Zonisamidum Neuraxpharm) r. Other
19	Was the epilepsy treated with CBD (cannabidiol)?	[Single choice]	a. Yes b. No
20	If so, what dosage and active substance concentration was used?	[Short answer]	–
21	Does the affected individual experience sleep disorders?	[Single choice]	a. Yes b. No
22	If so, has any treatment for these disorders been implemented?	[Short answer]	–
23	Has the affected individual been to a gastroenterologist?	[Single choice]	a. Yes b. No
24	Has the affected individual ever experienced reflux?	[Single choice]	a. Yes b. No
25	Does/did the affected individual experience constipation?	[Single choice]	a. Yes b. No
26	If eating disorders were present, was any treatment introduced? If so, what kind?	[Short answer]	–
27	Does the affected individual experience a noticeably increased appetite (hyperphagia)?	[Single choice]	a. Yes b. No
28	Has the affected individual ever been, or are they currently, on a ketogenic diet?	[Single choice]	a. Yes b. No
29	Has the affected individual ever been, or are they currently, on a low GI (Glycemic Index) diet?	[Single choice]	a. Yes b. No
30	Is the affected individual currently on any prescribed diet?	[Short answer]	–
31	Are any spinal or limb abnormalities present in the affected individual?	[Single choice]	a. Yes b. No
32	Is the affected individual under the supervision of an orthopaedist?	[Single choice]	a. Yes b. No
33	Is the affected individual undergoing rehabilitation?	[Single choice]	a. Yes b. No
34	If so, what kind of rehabilitation is it? (currently)	[Short answer]	–
35	If the affected individual is undergoing rehabilitation, please state the age at which this rehabilitation began. (age in months)	[Short answer]	–
36	Has water-based rehabilitation been, or is it currently being, implemented?	[Single choice]	a. Yes b. No
37	Is the affected individual undergoing speech therapy?	[Single choice]	a. Yes b. No
38	If so, what kind of speech therapy is it? (currently)	[Short answer]	–
39	If the affected individual is undergoing speech therapy, at what age did this therapy begin? (age in months)	[Short answer]	–
40	Does the affected individual communicate verbally (i.e. through use of words)? If so, how many words are used and since what age? (please provide an estimate, e.g. 5 words, 10–15 words, as well as the age of the first utterance in months)	[Short answer]	–
41	Are the affected individual’s family members under the care of a psychologist/therapist?	[Single choice]	a. Yes b. No
42	Does the affected individual experience any kind of visual impairment?	[Single choice]	a. Yes b. No
43	If so, what kind and to what degree? (e.g. myopia/hyperopia/astigmatism, if possible please specify the impairment in diopters/dioptres)	[Short answer]	–
44	If visual impairment is present, at what age was it diagnosed? (age in months)	[Short answer]	–
45	What do you consider to be the biggest problem/obstacle in the diagnosis of Angelman Syndrome in Poland? (please state one aspect that you found most difficult when seeking to obtaining a diagnosis)	[Short answer]	–
46	What do you consider to be the biggest problem/obstacle in the treatment of Angelman Syndrome? (please state one aspect that you find most difficult)	[Short answer]	–

## Results

**Fig. 1:**
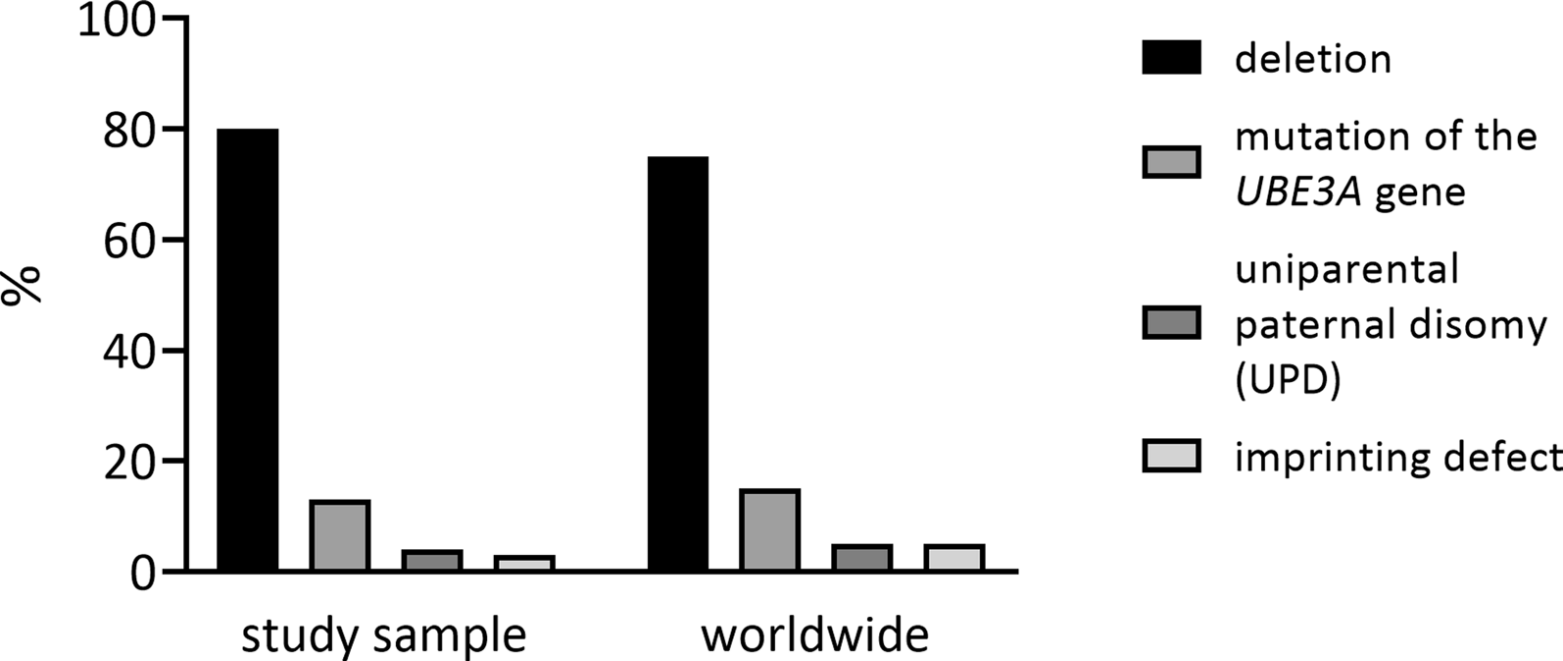
Comparison of the prevalence of different genetic background in the study sample and worldwide (according to Duis et al. 2022 [1])

In the single-choice question: "Which genetic background for Angelman syndrome was identified during the diagnosis?", out of five possible answers: 80% of caregivers (n = 56) indicated a deletion (of the maternal region containing *UBE3A*), 12.9% a mutation of the *UBE3A* gene, 4.3% paternal uniparental disomy (UPD), and 2.8% an imprinting defect. None of the respondents indicated a clinical diagnosis (based on symptoms) [Fig. 1].

The median (Me) age of the study sample group is 60 months, and the mean (M) is 79 months, as the youngest group are patients with deletion (Me = 57 months, M = 66 months). The first symptoms appeared at an average age of 5 months (Me = 5 months).

**Fig. 2:**
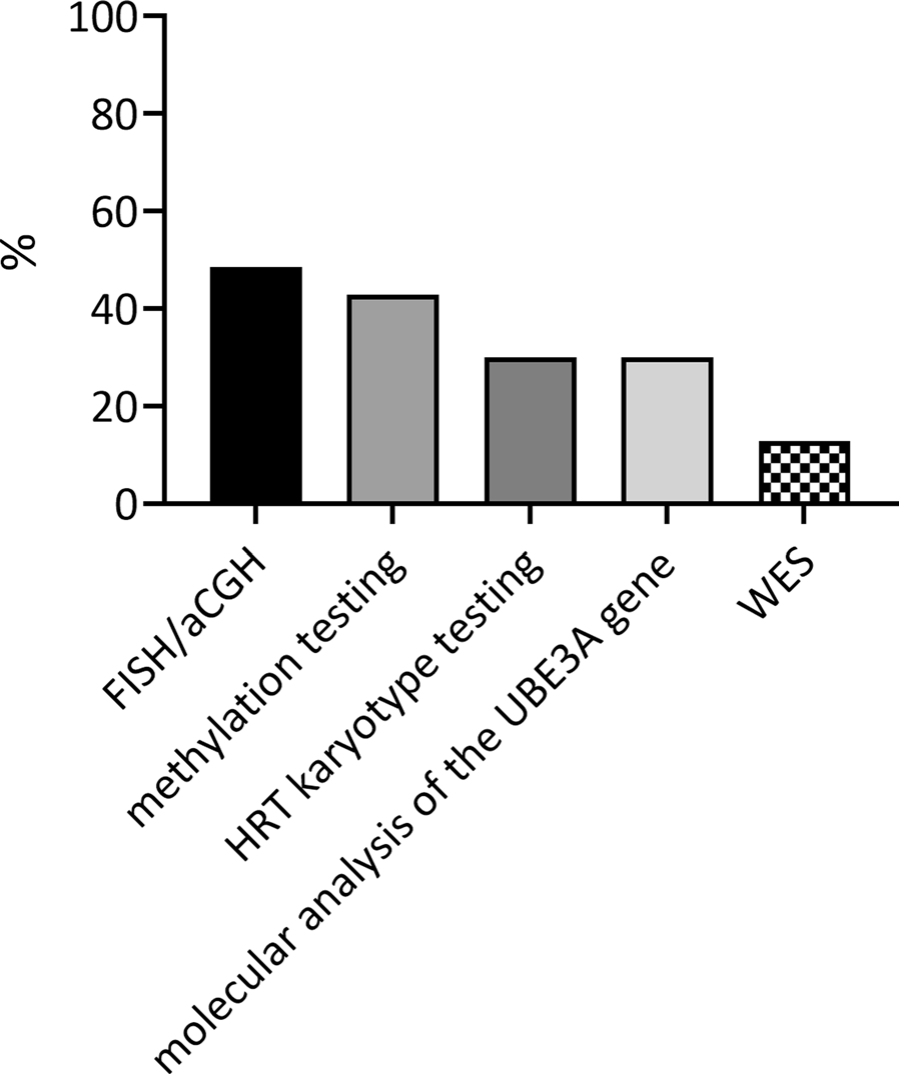
Genetic tests performed during the diagnostics. Abbreviations: FISH fluorescence in situ hybridization; aCGH array comparative genomic hybridization; whole exome sequencing (WES). (*Note*: It was a multiple-choice question, so every participant could select multiple answers.)

In a multiple selection multiple choice question regarding the presence of the first worrying symptoms, the caregivers most frequently indicated: delayed motor development (n = 63), abnormal muscle tension (n = 45), sucking problems (n = 35), delayed intellectual development (n = 29), and speech disorders (n = 27). The behavioral phenotype of AS patients, represented by unexpected laughter and general inappropriate behavior, was described as a significant problem for parents of 15 and 13 children, respectively. In a single choice question, the caregivers indicated that the mentioned symptoms were first noticed by caregivers (67%), followed by medical professionals (17%). In 16% of the surveyed families, concern about global development and the suggestion of a genetically determined syndrome were noticed during hospitalization due to delayed psychomotor development. The median onset of diagnosis was 12 months (M = 18 months)—the earliest in patients with deletion (Me = 11 months, M = 14 months). At diagnosis, FISH or aCGH microarray testing was most commonly performed (n = 34; 48.6%) of patients, followed by methylation testing (n = 30; 42.9%), maternal and child HRT karyotype testing (n = 21; 30%), molecular analysis of the *UBE3A* gene (n = 21; 30%), and *Whole Exome Sequencing* (WES) testing (n = 9; 12.9%) [Fig. 2].

**Fig. 3:**
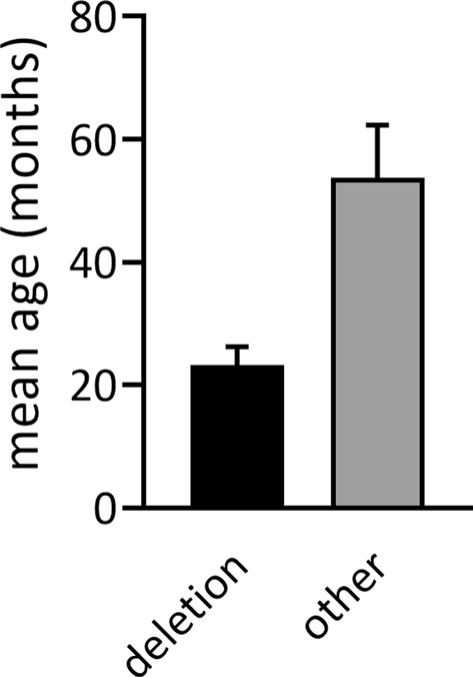
Mean age of diagnosis depending on genetic background (deletion vs. other backgrounds). Data presented as mean ± SEM

The mean duration of diagnosis was 14 months (Me = 7 months). The mean age of diagnosis of AS in the group of AS patients was 29 months, or 2.4 years (Me = 18.5 months). The earliest diagnosis occurred in the subgroup with deletion—after nearly 2 years (M = 23 months; Me = 18 months). In the group with a background other than deletion, the mean diagnosis was 53.7 months, or 4.5 years (Me = 47.5 months) [Fig. 3].

The median waiting time to see a clinical geneticist was 3 months (M = 4.6 months). More than half of the caregivers (51.5%) incurred additional costs during the diagnostic process, on average 3900 PLN (= 930$), Me = 2350 PLN (= 560$).

During the diagnostic process, 37.9% of patients received a different diagnosis before the diagnosis of AS. One-fifth (20%) of the misdiagnoses were ones of cerebral palsy, which was most frequently diagnosed in the group of patients with the *UBE3A* mutation—37.5%. In contrast, it was least frequently diagnosed in patients with a deletion—17%. Other diagnoses that appeared in the diagnostic process were West syndrome, Cri-du-Chat syndrome (5p deletion syndrome), spinal muscular atrophy (SMA), Dravet syndrome, or PWS.

The reassessment of a patient's condition due to a prior misdiagnosis has a negative impact on the time to initiate appropriate treatment. While the average time to diagnosis was 10 months in case of children without another diagnosis, prior misdiagnosis prolonged this time more than twice on average (M = 22 months).

Almost every child (98.6%) is examined, at least once every 6 months, by a pediatric neurologist. Among the neurological symptoms, caregivers mention epileptic seizures, which appear at an average age of 27 months (Me = 24 months) and occur in 88.6% of the sample group. In the study group, epilepsy was most common in patients with deletion—91% (epileptic seizures appeared at 26 months on average; Me = 24 months). The most commonly used drugs were valproic acid, levetiracetam, clobazam, pregabalin, and clonazepam. Vigabatrin was used in 8.6% of subjects (n = 6).

In 8.6% of the study population, cannabidiol (CBD) was used in daily therapy, with a mean dose of 2.5 mg/kg/days.

Sleep disorders are present in 78.6% of patients (n = 55). Interestingly, its severity is similar among groups with different genetic backgrounds. Half of these individuals use pharmacological treatment for sleep disorders. A short-answer question revealed that 11% (n = 6) of people use both melatonin and other substances such as risperidone, hydroxyzine, clonazepam, nitrazepam, dimethindene, or herbal preparations such as lemon balm (*melissa officinalis*) as therapeutic agents. Melatonin as the only treatment is used by 24% (n = 13) of patients, while other substances (such as those mentioned above) are used by 15% (n = 8).

A consultation with gastroenterologist was made by 47.1% of patients, most often in the UPD group with a ratio of 67%. More than half of the patients (n = 39; 55.7%) with AS experienced pathological gastro-esophageal reflux disease (GERD). This disease was most common in the group with the *UBE3A* mutation—67% of individuals. Constipation was present in 71.4% of patients (n = 50), most commonly in the group with the deletion—75% of individuals. 27.1% of the children of individuals (n = 19) had hyperphagia, most commonly in the group with UPD*—*67% of individuals.

A low-carbohydrate diet is used in 12.9% of patients (n = 9), including a ketogenic diet in 7.1% of individuals, most commonly in patients with deletion (9%).

Among the study group, 61.4% (n = 43) have spinal or limb defects, 50% are under the supervision of an orthopedic surgeon, and rehabilitation has been carried out in 98.6% of patients and on average since 7.9 months of age (Me = 5.5 months). Rehabilitation was started earliest in the UPD group, with a mean of 3.7 months (Me = 3 months). The most frequently used types of rehabilitation (multiple selection multiple choice question) were NDT-Bobath—52%, Vojta—27.5%, speech therapy—23.2%, sensory integration method—21.7%, motor rehabilitation—18.8%, and Medek method—7.2%. More than one type of physiotherapy is used in 58.6% of respondents, and 54.3% undergo water therapy.

Of the individuals surveyed, 43.6% (n = 24) communicate verbally and have spoken an average of 5 words since the age of 26 months. Verbal communication has been found to be most infrequent among the patients with deletion—33%. It is also these patients that speak the least as measured by number of words, the median on this measure being 3. Speech therapy is provided to 71.4% (n = 50) of people. In a multiple selection multiple-choice question, respondents indicated that the most frequently used methods are assistive and alternative communication (AAC)—52% and work with a speech therapist—30%. Speech therapy is provided on average from 29 months of age (Me = 24.5 months).

Visual impairment is present in 57.1% of patients (n = 40), of whom 34.2% have astigmatism, 22.9% hyperopia, and 10% myopia. 15.7% of respondents report the children under their care having strabismus, and 2.9% report lensless. Visual defects were diagnosed at an average of 30 months of age (Me = 24 months).

11.6% respondents (n = 8) reported that at least one of their family members sought psychological support.

The survey also included open questions for caregivers, such as:—"What do you consider to be the biggest problem in the diagnosis of Angelman syndrome in Poland?". 64.3% (n = 45) of respondents indicated that the biggest problem is finding the right specialist to notice abnormalities and refer the child for detailed genetic tests. Another impediment is hampered access to specialists experienced in treating people with AS. Respondents also point out a lack of sufficient knowledge about rare diseases among health care professionals. Some respondents (38.6%; n = 27) also report problems with the availability of tests and long wait times before receiving results, both factors contributing to delays in diagnosis.

## Discussion

### Genetic structure of the population

The genetic background for the prevalence of AS in our sample group [Fig. 1] matches the distribution reported by Duis et al. in the consensus report on standards of care for patients with AS [1]. Patients with a deletion of the maternal region 15q11.2q13 constitute the largest group, amounting to 80% compared to 70–75% of individuals reported by the consensus. The percentage of unique AS substrates also corresponds with the results of a study by Du et al. on a large Chinese cohort [11]. Therefore, we assume that the group involved in our study is a representative sample of patients with AS.

### Population age analysis

AS in the study group was diagnosed earliest in patients with deletion. Thus, these patients present the most severe clinical manifestation of the AS and are the fastest to be referred for specialized investigations. Those results correlate with data from other studies [4, 5, 12]. The mean age of diagnosis, for the general group, is higher than that reported by Khan et al. in the Angelman Syndrome Natural History Study (2 years vs. 2.4 years in the presented study) [13]. This age is also higher than the mean age at the time of diagnosis in the Dutch population based on the work of Bindels-de Heus et al. (22.5 months vs. 29 months in the presented study) [10]. There is also a larger discrepancy in the comparative analysis of the group of patients with a genetic background other than deletion. The mean age of diagnosis for non-deletion reported by Khan et al. is 2.9 years (vs. 4.5 years in the presented study) and 33.8 months (vs. 53.7 months in the presented study) [10, 13]. The cited differences illustrate the significant diagnostic difficulties experienced by patients with AS in Poland, leading to a delay in making a correct diagnosis, especially in the group of patients with less specific symptoms.

### First symptoms

The first symptoms of AS most often reported by caregivers, such as delayed motor development, abnormal muscle tone or sucking problems, are consistent with the data available in the literature. In the neonatal and infant period, these symptoms are non-specific [5, 14, 15]. Symptoms related to the behavioral phenotype (unexpected laughter, inappropriate behavior) were reported by a smaller proportion of caregivers.

### Diagnostic testing

Although genetic diagnostics in Poland is available at an advanced level, unfortunately, this access is hampered by the lack of reimbursement for some genetic tests, especially broad-spectrum tests, and the lack of publicly available information on the location of reference genetic laboratories. Such information should be brought together on a information platform run by specialists and covering all rare genetic diseases. There is usually a long period of time between the detection of the first worrying symptoms by parents (Me = 5 months) and referral for genetic testing (Me = 12 months). However, early diagnosis of AS is one of the key factors that significantly improves prognosis and the long-term effect of therapy. The timing of early initiation of speech therapy and rehabilitation seems to be particularly important [16]. The basic characteristics of AS should be more widely disseminated among pediatricians and other health care professionals in order to improve standards regarding the diagnosis and treatment process, as well as the quality of life of patients and their families. Furthermore, there is a general lack of knowledge on rare diseases in the Polish medical community, which necessitates the provision of additional training to pediatric and family medicine specialists and other health care professionals [8].

#### The WES study

The WES test is not recommended as a first-line test for the diagnosis of AS [1]. Despite this, a WES test was performed in 12.9% of the patients in the study group. The diagnostic procedure is associated with high financial costs for families and is often promoted by commercial diagnostic companies or social media groups for families of children with and without the diagnosis of a disorder. -As we have already mentioned above, this might be due to the difficulty in accessing reliable information, for example, the lack of a Rare Disease Information Platform. According to the recommendations, the genetic background of AS can be investigated using methods that are endorsed by geneticists and reimbursed, such as FISH, aCGH microarray, or molecular analysis of the *UBE3A* gene sequence. WES testing should be reserved for rare cases with diagnostic difficulties that cannot be detected by other molecular methods [17, 18]. The WES test cannot unambiguously assess the most common cause of AS, which is abnormalities in the methylation pattern of the critical 15q11-q13 region associated with AS. In addition, the WES test may reveal a number of non-specific variants that are unrelated to the child's underlying developmental problems [19, 20].

### Incorrect diagnosis

Early diagnosis of AS is crucial for improving the prognosis and achieving satisfactory treatment outcomes for patients [16]. It is important that a correct diagnosis of AS is not preceded by another diagnosis. Unfortunately, this was quite frequently the case in the presented study group. As many as 37.9% of the patients had received an incorrect pre-diagnosis. One-fifth of the patients (20%) had previously been diagnosed with cerebral palsy (CP), mostly among the *UBE3A* mutation group, which may be due to the mildest clinical presentation of patients with this genetic background [4, 5, 21]. On the other hand, the smallest group of misdiagnoses were patients with deletion, which is also consistent with the study by Roche et al.—this phenotype is the most pathologically altered [22]. The diagnosis of CP should be followed by a broader differential diagnosis and exclusion of other conditions. Most of the misdiagnoses in the study group correspond with the data available in the literature, including PWS or Rett syndrome [5, 22]. However, there were also new faulty diagnoses, such as: SMA, Dravet syndrome or 5p deletion syndrome (Cri-du-Chat). It is worth to point out that the number of misdiagnoses in the Polish population is lower than the 48% reported by the Global Angelman Syndrome Registry [22], which might be observed due to limited sample size of our study.

### Epileptic seizures

According to the recommendations, almost all patients are under the supervision of a pediatric neurologist [1, 5]. The vast majority of patients have epileptic seizures, with the earliest appearance at 27 months in patients with disease caused by deletion. This is consistent with available data on the most severe phenotype in patients with this genetic background [12].

Although the majority of the patients in the study group were treated with the recommended medications, 8.6% have been reported to be treated using vigabatrin, which is not recommended in AS [1, 23]. It might be an effect of the current survey for caregivers in which the correlation between the diagnosis of epilepsy and the initiation of vigabatrin therapy was not investigated. It is not uncommon for a patient to present a flexion seizure morphology before the molecular diagnosis of AS, which corresponds to the clinical diagnosis of West syndrome, in which vigabatrin is the dedicated drug. On the other hand, the lack of knowledge of current recommendations for the treatment of epilepsy in AS in the pediatric neurology community must be taken into account. It may indicate the need for separate centers for patients with AS. This approach is applied in many countries, such as the United States of America, Netherlands, Germany, Spain and Israel and helps to avoid therapeutic errors in children with AS.

### Use of cannabinoids (CBD)

Few patients (8.57%) were treated with *CBD*. Analysis of acquired data showed that the prescribed dosage was significantly lower (2.5 mg/kg/day on average) than the effective doses recommended in the treatment of other conditions (10 to 20 mg/kg/day [24]), such as Lennox-Gastaut syndrome, Dravet syndrome, and tuberous sclerosis. Furthermore, in a clinical trial of patients with AS conducted by *Radius Pharmaceuticals,* the dose was set even higher at 25 mg/kg/day (conference materials—SCOUT1-019 trial). Thus, we can conclude that the dosage administered to the patients in the group described here was significantly smaller (10 times). Probably there was no therapeutic effect, such as a reduction in epileptic seizures, or improvement in cognitive function.

### Sleep disorders

Sleep disorders are one of the most troublesome symptoms present in the course of AS, posing a problem for the whole family living with the patient. Sleep–wake disorders are present in 78.6% of the study group, with no differences between distinct genetic backgrounds; those data are consistent with previous reports [25–28]. Melatonin was used in only 39% of patients, although according to recommendations, it should be used for sleep disorders in patients with AS [1]. Other substances approved for short-term use (e.g.: benzodiazepines) were used in 27% of the patients, though the questionnaire did not involve an analysis of the duration of benzodiazepine therapy. Other medications (risperidone, hydroxyzine, dimethindene) used for the treatment of sleep disorders seem controversial and, as with the use of vigabatrin in patients with AS, demonstrate the need for education of neurologists and child psychiatrists, confirming the need for referral centers for AS [1, 5].

### Gastroenterological problems

Gastroenterological disorders in people with AS are very common, they affect nearly every patient [29]. The complaints can vary, including constipation, infant feeding problems, diarrhea, gastroesophageal reflux disease (GERD), and cyclic vomiting [29, 30]. The aforementioned difficulties were also present in the study group. According to recommendations, every patient diagnosed with AS should be under the supervision of a gastroenterologist [1, 14], but only 47.1% of the study group have had a consultation with a specialist. It is particularly important as gastroenterological problems are associated with other complaints such as hyperhidrosis during the sleep and the rate of nocturnal urinary continence [31].

### Hyperphagia

Hyperphagia is more often associated not only with PWS but also with AS [32, 33]. It is crucial for clinicians not to equate excessive appetite and obesity with PWS, as this can lead to misdiagnosis. It is also essential knowledge for caregivers, given their responsibility for preparing meals that should prioritize higher protein content over carbohydrates. The patient’s body weight should be monitored by a general practitioner, and abnormal results should be consulted with specialists [1, 5].

Hyperphagia was present in 27.1% of the study population, which corresponds with the results of other studies indicating that the affliction affects approximately one-third of AS patients [10, 32–34]. In the study group, hyperphagia was most common in patients with *UPD* (67%). It is consistent with the results of a study conducted on a Danish cohort, the percentage of hyperphagia was also higher among patients with UPD*.* The researchers posited the hypothesis that an increase in body weight is associated with an escalation in paternal gene copy expression [34]. A low-carbohydrate diet (ketogenic diet or low glycemic index diet) is only used in 12.9% of patients, although its effectiveness has been shown in reducing the severity of epileptic seizures in people with AS (especially in cases that present with drug resistance). This confirms the need to educate the pediatric neurology community on the treatment of drug-resistant epilepsy in AS [1, 35].

### Visual impairment

Visual impairments are present in 57.1% of the study group, among which astigmatism is particularly frequently diagnosed. However, comparing the results with the study performed by Michieletto et al. the percentage of astigmatism appears to be significantly lower (34% vs. 94% in the Italian group). There is also lower percentage of hyperopia and strabismus—22.9% versus 76% and 15.7% versus 75% respectively. The proportion of myopia is similar in both groups (10% vs. 9%) [36]. The discrepancies mentioned above may indicate that access to ophthalmic and orthoptic consultation is limited for the study group.

### Rehabilitation

Spinal and limb defects are common in people with AS, with 61.4% of the sample group affected [37, 38]. Although guidelines recommend a yearly follow-up examinations of developmental progress in the area of mobility, only 50% of patients are under the supervision of an orthopedic surgeon [5, 39, 40].

Individually tailored therapies depending on the needs of the individual patient are crucial. The benefits of early and appropriate care cannot be overstated and include the prevention of such complications as joint contractures and progression of scoliosis [3, 41–43]. The median onset of rehabilitation among the study population is 5.5 months, which seems to be a good result, while no data are available for other populations.

Among the studied group, in almost all cases (98.6%), rehabilitation is conducted. Different methods are used—NDT-Bobath, speech therapy, Vojta therapy, sensory integration, or Medek therapy. Currently, there are various methods of physiotherapy treatment for Angelman syndrome and there is no uniform approach. This is caused by a variety of individual symptoms with varying degrees of intensity. Based on a systematic review of the literature, it cannot currently be concluded that one physiotherapy method is more effective than another [44]. In such situations, it is always recommended to use as many different therapies as possible, adapted to the current clinical condition of the patients. Therapeutic methods used for similar psychomotor, motor, functional and communication disorders should be used in this regard. Therapies based on improving the coordination of movements and reflex reactions using the Vojta and NDT-Bobath methods improve the central coordination disorder, which occurs as a symptom of AS and are also enhancing gross motor performance [43, 45]. Also a number of methods based on the stimulation of equivalent static and dynamic reactions, which, in addition to improving motor functions, including walking, reduce the number of falls [46].

There are also treatment methods for AS that have no evidence of effectiveness in current research; hippotherapy, cranial osteopathy, aromatherapy, reflexology, hydrotherapy, music therapy, brushing, static cycling, and trikes [47]. None of our participants has reported using those methods.

A considerable proportion of patients (54.3%) participate in water therapy. This is particularly recommended because of the interest AS children exhibit towards water. Research has shown that this is a stimulus that can increase the effectiveness of the provided therapy [15].

The next direction should be the search for new physiotherapeutic methods that will be very specifically tailored to reducing AS symptoms. One of these methods may be neuromobilization of the peripheral and central nervous system—stimulation of the nervi nervorum, and of the vagus nerve—stimulation of interoreceptors in order to trigger the process of brain plasticity [48].

Future research in this area should be conducted towards the comparison of various methods, or rather towards the development of criteria for the inclusion of different methods depending on the current clinical symptoms. In summary of this section, physiotherapy carried out fully according to recommendations [1].

### Speech therapy

It is important to carry out an early assessment of motor, sensory, cognitive, and language abilities to ensure that the patient develops the above skills in the first stages of life [49]. Within the group surveyed in the present study, 78.4% of patients are receiving speech therapy. Caregivers report 29.3 months (median 24.5 months) as the time when therapy began. The most commonly used form of therapy is AAC alongside work with a speech therapist. Within the studied group, 43.6% of people with AS communicate verbally. They speak an average of 4.8 words (types) as the majority of our study group is diagnosed with deletion, which is consistent with previous findings [38, 49, 50]. In contrast, people with the mosaic form of AS are able to use short sentences using up to 60 words [51, 52]. The mean age for verbal communication in the study group is 26.6 months (median 24 months) in contrast to the information in the article prepared by Buntinx et al., who report that the first word that is frequently used out of context can occur between 10 and 18 months of age [50].

### Psychological support

The occurrence of a rare disease in a family often experience negative impact on the psychological well-being of its individual members [53]. A comparative study found that parents of children with AS experience the highest levels of stress. Mothers are particularly impacted [54], which may be due to the fact that they are the ones who most often serve as primary caregivers to the affected child [55]. The literature also highlights the potentially negative impact of numerous stressors on the family's social life [1, 56]. In research studies, the majority of parents of children with rare diseases indicate the need to receive psychological support [57], while in the study group, only 11.6% reported receiving such support. The results point to the importance of providing extended psychological support to entire families of children with a rare disease, this would include caregivers, siblings, and the affected children themselves [58]. This approach would support their functioning through a range of beneficial interactions in response to individual needs.

### Comments from parents

The biggest difficulties reported by caregivers are finding an appropriate specialist and the lack of sufficient knowledge about rare diseases among healthcare professionals in Poland. These results are consistent with the study by Walkowiak et al., which highlighted the lack of knowledge in the area of rare diseases and pointed out the problem of the need to effectively differentiate these diseases from the more commonplace ones. Additional courses for medical professionals should be provided to fill the gap in current knowledge [8]. Black et al. created a report on the diagnostic odyssey in rare diseases, and presented solutions which include the creation of special registers at the level of national or individual centers, in which the time of spotting individual symptoms by caregivers would be reported [59]. Another solution could be the creation of a system where children with developmental abnormalities that occur during a check-up are referred for specialized testing. Such a solution would be beneficial for pediatricians working in primary care practices, as it would not be their responsibility to diagnose rare diseases in detail but to notice abnormalities during a basic examination. In addition, we recommend creating specialized clinics to treat rare diseases and a database of specialists so that those seeking help can get the necessary support as soon as possible.

### Limitations

Due to the size of the study group and the distribution of the genetic background in the AS population, available sub-groups are not matched in terms of size. The characteristics of the depth of the disorder according to the type of genetic background are significantly different [1]. Due to the small size of the individual sub-groups, we could not perform standard statistical tests because their results would be subject to high error rates. The survey was anonymized and conducted online. The analysis included responses from caregivers of children with a diagnosis of *AS*, most of whom have no medical background. The data obtained were not verified against the patients' medical records.

## Conclusions

Specialist care for patients with AS in Poland does not differ significantly from European and world standards, with the main difference being access to specialists in clinical genetics and the verification of who requires specialist genetic diagnostics. That said, physicians in primary healthcare have been reported to lack appropriate knowledge about rare diseases [8]. Moreover, access to specialists dedicated to patients with AS is very limited, care is not coordinated, and specialists have to be sought by caregivers on their own. Given this state of affairs, it would be advisable to create specialized centers or databases of specialists (referral centers dedicated to patients with rare diseases, including patients with AS) that could support families in need. In addition, the creation of a dedicated for polish patients Rare Diseases Information Platform could be helpful. While this step is featured in the Rare Diseases Plan (“Plan dla chorób rzadkich”) adopted by the government, it has not yet been implemented. Finally, the role of psychological support should not be overlooked in the care protocols dedicated to patients with AS and their families.

## Data Availability

The datasets used and/or analysed during the current study are available from the corresponding author on reasonable request.
